# Neurodevelopmental risks of late-preterm and early-term births: a population-based study from Finland

**DOI:** 10.1136/bmjph-2025-003708

**Published:** 2026-02-27

**Authors:** Samson Nivins, Mika Gissler, Catharina Lavebratt

**Affiliations:** 1 Department of Women's and Children's Health, karolinska Institutet, Stockholm, Sweden; 2 Department of Data and Analytics, Finnish Institute for Health and Welfare, Helsinki, Finland; 3 Center for Molecular Medicine, Karolinska University Hospital, Stockholm, Sweden; 4 Academic Primary Health Care Centre, Region Stockholm, Stockholm, Sweden; 5 Department of Molecular Medicine and Surgery, Karolinska Institutet, Stockholm, Sweden

**Keywords:** Epidemiology, Mental Health, Adolescent, Sex Factors

## Abstract

**Background:**

Being born late-preterm and early-term is a known risk factor for neurodevelopmental disorders; however, most studies have focused on intellectual disabilities (ID), autism spectrum disorder (ASD), and attention-deficit/hyperactivity disorder (ADHD), with limited attention to diagnostically distinct disorders of communication or motor development. It remains unclear whether these associations are directly attributed to earlier birth or confounded by shared genetic and environmental factors.

**Method:**

This population-based cohort study included over 1 million singleton children born between 34^+0^ and 40^+6^ weeks of gestation in Finland during 1996 and 2014, with follow-up for neurodevelopmental disorders through December 2021. Cox proportional hazards models estimated associations between gestational age at birth and risk for neurodevelopmental disorders, adjusting for confounders. Sibling-pair analyses assessed the influence of unmeasured shared familial factors.

**Results:**

Of the cohort, 2.0% were born at 34^+0^ to 35^+6^ weeks, 7.8% at 36^+0^ to 37^+6^ weeks, 13% at 38 weeks and 76.5% between 39^+0^ and 40^+6^ weeks. Prevalence of neurodevelopmental disorders declined with increasing gestational age: 21.6% at 34^+0^ to 35^+6^ weeks, 19% at 36^+0^ to 37^+6^ weeks, 16% at 38^+0^ to 38^+6^ weeks and 15% at 39^+0^ to 40^+6^ weeks (full-term). Compared with full-term, children born between 34^+0^ and 38^+6^ weeks had a higher risk of communication disorders, motor disorders and specific learning disorders, with the greatest risks among late-preterm births; risk estimates were similar across sexes. These associations persisted after adjustment for potential confounders, including familial factors shared between siblings. Risks for ID, ASD and ADHD were also observed.

**Conclusion:**

Being born late-preterm and early-term is associated with higher risks of neurodevelopmental disorders. These groups have traditionally received less attention, often being considered at low risk. Our findings underscore the importance of recognising that even late-preterm and early-term births carry measurable neurodevelopmental risk. Promoting longer gestation may support more optimal neurodevelopmental outcomes.

**WHAT IS ALREADY KNOWN ON THIS TOPIC**

- The risk of childhood neurodevelopmental disorders such as intellectual disabilities, autism spectrum disorder, and attention-deficit/hyperactivity disorder is lower with higher gestational age. These associations are similar in boys and girls and appear largely independent of perinatal, maternal and shared familial factors.

**WHAT THIS STUDY ADDS**

- Being born late-preterm or early-term, even at 38 weeks, is associated with higher risks of the more common communication, motor and specific learning disorders. These risks persist after accounting for environmental and shared familial factors, raising concerns about current gestational age thresholds for low-risk classification.

**HOW THIS STUDY MIGHT AFFECT RESEARCH, PRACTICE OR POLICY**

- Motor, communication, and specific learning disorders are highly prevalent but often under-recognised in children born beyond 32 weeks. These findings highlight the need for greater clinical attention to early identification and support, and underscore the importance of avoiding non-medically indicated early births to optimise neurodevelopmental outcomes.

## Introduction

 Preterm birth (<37 weeks) is a known risk factor for neurodevelopmental disorders throughout childhood and adolescence^1^,^5^ and represents a growing public health concern globally. As the risk of adverse outcomes increases progressively with lower gestational age, most researchers have concentrated on the highest-risk population, namely those born before 32 weeks, who account for 1%–2% of all births.^6^

Of all preterm births, ~70% are late-preterm (34^+0^ and 36^+6^ weeks).^7 8^ Although morbidity and mortality rates in this group are relatively low, there has been growing focus over the past decade on their significance, owing to the large number of births and their substantial contribution to healthcare service needs and costs. For example, children born late-preterm are at a higher risk of acute respiratory disorders immediately after birth and delayed feeding development compared with their full-term peers (39^+0^ and 41^+6^ weeks).^9 10^ Similarly, mounting evidence suggests that children born early-term (37^+0^ and 38^+6^ weeks) are also at higher risk of neonatal respiratory morbidity and neonatal intensive care unit (NICU) admissions compared with those born full-term.^11^

Beyond short-term morbidities, children born late-preterm or early-term are also at higher risk of developing neurodevelopmental disorders, primarily evident during childhood and adolescence. A small but growing number of population-based registry studies have examined this association, shedding light on the long-term neurodevelopmental consequences of being born before full-term.^412^,^18^ Notably, some of these studies have employed familial designs to help disentangle genetic from environmental contributions, an important consideration given the strong heritability of many neurodevelopmental disorders. Their findings suggest that a higher risk associated with late-preterm and early-term birth is largely independent of shared familial factors.^4 12 13 17^ However, existing studies have mainly focused on diagnoses such as intellectual disabilities (ID), autism spectrum disorder (ASD), and attention-deficit/hyperactivity disorder (ADHD), with limited attention to other neurodevelopmental disorders of childhood.

Studies have shown that late-preterm or early term births are associated with difficulties in communication and motor domains and tend to perform more poorly in school.^19^,^22^ For example, the UK Millennium Cohort Study (n=6031) found that late-preterm children had significantly poorer performance in reading and writing, with risk ratios ranging from 1.35 to 1.55 compared with their full-term peers.^22^ At the same time, some studies have reported no association.^23^ These inconsistencies emphasise the need for large population-based studies to better quantify the risk associated with being born late-preterm or early-term. Further, it remains unclear whether these associations are due to gestational age per se or confounded by shared genetic and environmental factors. Due to the high prevalence of late-preterm or early-term births, impaired neurodevelopmental outcomes may translate into a considerate public health burden.

To address this gap, we conducted a population-based cohort study including over 1 million children in Finland. Our primary aim was to examine the association between gestational age at birth (34^+0^ to 40^+6^ weeks) and the risk of neurodevelopmental disorders during childhood and adolescence. Given the high prevalence of neurodevelopmental disorders in boys compared with girls, we also explored the sex differences in these associations. Further, we investigated the extent to which shared genetic and environmental factors might account for the observed associations.

## Methods

We included all liveborn singleton children in Finland between 1996 and 2014, born at gestational age 34^+0^ to 40^+6^ weeks with a valid personnel identification number, given to all Finnish citizens and permanent residents, enabling linkage across national health and population registers (n=1 036 664), with follow-up until December 2021. These registries included the Medical Birth Register and Finnish Care Registers for Health Care. Ethical approval was obtained from the relevant data protection authorities and ethics committees in both Finland and Sweden (Sweden: 2023–03041 and Findata: THL/3922/14.06.00/2022, THL/5160/14.06.00/2023, THL/5391/14.02.00/2022 and THL/5159/14.06.00/2023). According to Finnish regulations, informed consent from participants was not required for register-based studies, and individuals were not contacted. This study adhered to the Strengthening the Reporting of Observational Studies in Epidemiology reporting guidelines. Data analysis was conducted between June and October 2024.

### Patient and public involvement

None. Patients and the public were not involved in the design, conduct, reporting or dissemination plans of this register-based study.

### Gestational age

Gestational age at birth was obtained from the Medical Birth Registry, primarily based on ultrasound estimates conducted at 11–13 weeks of gestation. The register also collects data on the last menstrual period, which is cross-checked against ultrasound estimates in cases of discrepancy between the two methods.

Given evidence from prior studies reporting dose-response relationship in neurodevelopmental disorders across the gestational age range of 34^+0^ to 38^+6^,^13 14^ we categorised gestational age into the following groups: 34^+0^ to 35^+6^ weeks, 36^+0^ to 37^+6^ weeks, 38^+0^ to 38^+6^ weeks and 39^+0^ to 40^+6^ weeks (reference group).

### Outcomes

Neurodevelopmental disorders were identified using International Statistical Classification of Diseases and Related Health Problems, 10th Revision (ICD-10) codes from the Finnish Care Register for Health Care, including both hospital inpatient and outpatient care. The following diagnostic categories were included: ID (F70–F79), communication disorders (F80), specific learning disorders (SLD; F81), motor disorders (F82), ASD (F84), ADHD (F90), conduct disorders (F91), and Tourette syndrome and other tic disorders (F95).

### Variables

Perinatal and demographic characteristics known to be associated with both gestational age at birth and neurodevelopmental disorders were identified from the Medical Birth Register and Finnish Care Register for Health Care (online supplemental eFigure 1). Maternal characteristics included maternal age at child’s birth (<20, 20–24, 25–29, 30–34 or ≥35 years), parity (nulliparous or multiparous), maternal occupation as a proxy for socioeconomic status (upper white-collar, lower white-collar, blue-collar or other), smoking during pregnancy (yes/no), hypertensive disorders (chronic hypertension, gestational hypertension or pre-eclampsia), diabetes (type 1, type 2 or gestational), body mass index (BMI; <18.5, 18.5–24.9, 25.0–29.9, 30.0–34.9 or ≥35), polycystic ovarian syndrome (PCOS; yes/no) and a history of psychiatric disorders (yes/no). Perinatal factors included mode of delivery (vaginal, instrumental, planned caesarean section or other caesarean section).

Child-related characteristics included birth size, categorised as small, appropriate or large for gestational age, based on sex- and gestational age-specific Finnish reference standards using a cut-off of two SDs below or above the mean, following the International Societies of Pediatric Endocrinology and the Growth Hormone Research Society.^24 25^ In addition, the child’s year of birth was included as a continuous variable to account for temporal changes in obstetric care and neurodevelopmental diagnoses over time.

### Statistical analysis

After confirming that the proportional hazards assumption was met, we used Cox proportional hazards regression models to estimate HRs and 95% CIs for the association between gestational age at birth and the risk of neurodevelopmental disorders during childhood and adolescence. This approach gives greater weight to cases with earlier age at onset within each outcome category, under the assumption that earlier diagnoses may reflect more severe conditions or outcomes less influenced by postnatal environmental exposures.

A series of models was fitted for each outcome, including both unadjusted (crude) and adjusted models. Model 1 adjusted for the child’s year of birth, parity, maternal age at child’s birth, socioeconomic status and mode of delivery. Model 2 included all variables from model 1, with additional adjustment for maternal smoking during pregnancy, maternal hypertensive disorders, maternal diabetes, maternal BMI, PCOS and maternal history of psychiatric disorders. Model 3 further adjusted for birth size. To assess whether early neonatal condition proxied by the 5-min Apgar score altered the estimates for the association between gestational age and the risk of neurodevelopmental disorders, model 4 included this variable. As we observed only minimal attenuation of associations across models, we present results from the crude model and models 3 and 4.

First, to examine whether risks of neurodevelopmental disorders varied across individual weeks of gestation (34^+0^ to 38^+6^ weeks), we conducted a week-by-week analysis using the covariates included in model 3. Based on the similarity of risk profiles across adjacent weeks, we grouped gestational age into broader categories (34^+0^ to 35^+6^, 36^+0^ to 37^+6^, 38^+0^ to 38^+6^ and 39^+0^ to 40^+6^ weeks) for subsequent analyses (online supplemental eResults and eTable 1). We then summarised maternal and child characteristics as counts and percentages (N and %) across these gestational age groups and conducted association analyses using grouped exposures in the unadjusted model and in models 1–3.

Given the large number of comparisons across gestational age groups and neurodevelopmental outcomes, we conducted a sensitivity analysis using model 3 in which HRs were estimated with 99% CIs for all gestational age groups and all neurodevelopmental disorders.

To investigate potential sex-specific effects on the association between gestational age at birth and neurodevelopmental disorders, we included a multiplicative interaction term between gestational age groups (34^+0^ to 35^+6^, 36^+0^ to 37^+6^ and 38^+0^ to 38^+6^) and sex in a crude Cox proportional hazards regression model. All interactions were statistically significant, supporting the presence of effect heterogeneity. We then conducted stratified analyses by sex (boys/girls) and compared effect estimates across boys and girls; these stratified analyses were conducted using model 3.

Further, to account for shared unmeasured familial confounding,^26^ we conducted a sibling-pair comparison model where pairs were stratified by their discordance or concordance for gestational age at birth. All sibling pairs from consecutive pregnancies were included. The risk of neurodevelopmental disorders in the second (younger) sibling was estimated by comparing three groups: pairs where only the second sibling was exposed, pairs where only the first sibling was exposed and pairs where both siblings were exposed. The reference group consisted of sibling pairs in which neither child was exposed (ie, both siblings were born at 39^+0^ to 40^+6^ weeks). The analyses were adjusted for the same covariates as in model 3 along with corresponding ICD-diagnosis related to mental and behavioural disorders in the first sibling (yes/no) and interpregnancy intervals (referred to here as model 5).

All statistical tests were two-sided, with an alpha level of 0.05. Analyses were performed using SAS V.9.4 (SAS Institute, Cary, North Carolina, USA).

## Results

**Figure 1:**
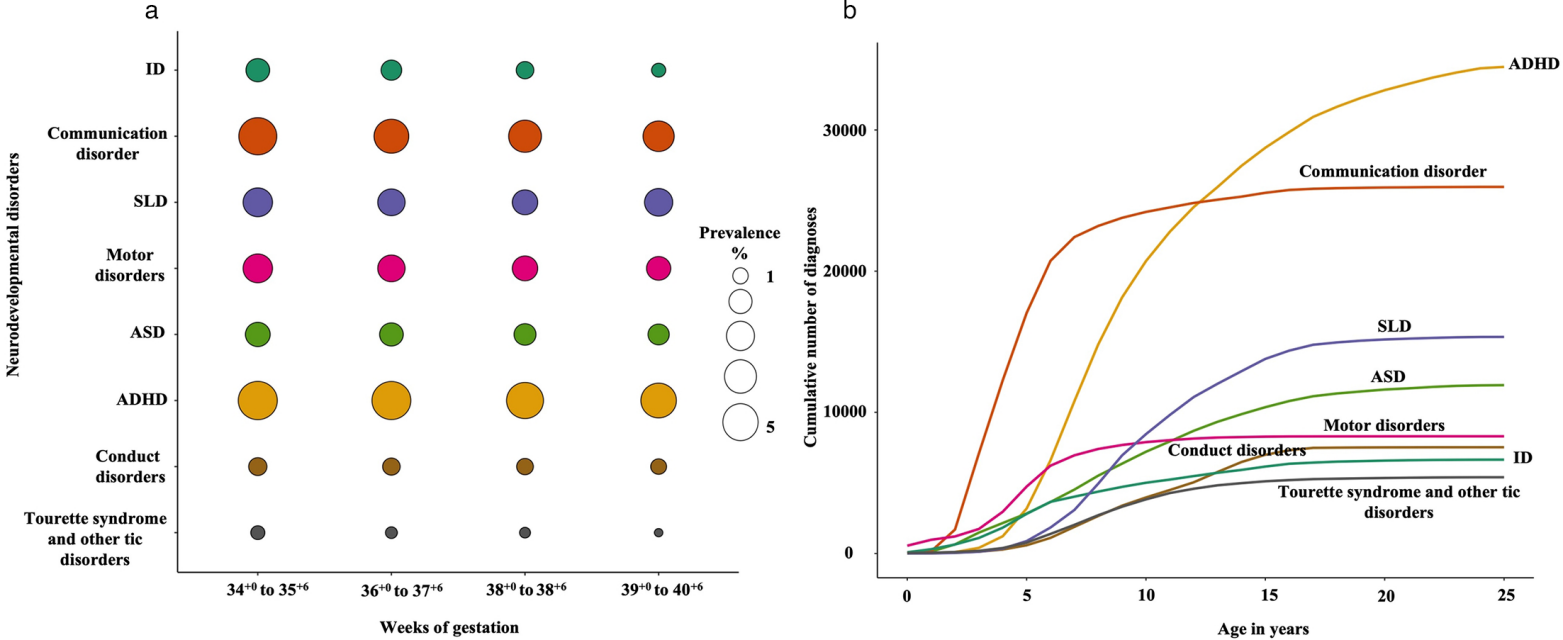
Prevalence of neurodevelopmental disorders among children born at different gestational ages in the Finnish population between 1996 and 2014. (**a**) Each bubble represents the prevalence of a given disorder, where the size and colour intensity of the bubbles correspond to the prevalence percentage with larger and darker bubbles reflecting higher prevalence. (**b**) The figure illustrates the cumulative number of diagnoses reported for each neurodevelopmental disorder studied across ages 0 to 25 years. Each coloured line represents the cumulative count for one diagnosis, allowing comparison of the age of onset and relative frequency over time. Intellectual disabilities (ID): F70–F79; communication disorders: F80; F81: specific learning disorders (SLD); F82: motor disorders; F84: autism spectrum disorder (ASD); F90: attention-deficit/hyperactivity disorder (ADHD); F91: conduct disorders and F95: Tourette syndrome and other tic disorders.

Between 1996 and 2014, 2.0% of children were born between 34^+0^ and 35^+6^ weeks, 7.8% between 36^+0^ and 37^+7^ weeks, 13% at 38 weeks and 76.5% between 39^+0^ and 40^+6^ weeks. The prevalence of any neurodevelopmental disorders was highest among those born at 34^+0^ to 35^+6^ weeks (21.6%) and decreased progressively with advancing gestational age, 19% at 36^+0^ to 37^+6^ weeks, 16% at 38^+0^ to 38^+6^ weeks and 15% at 39^+0^ to 40^+6^ weeks (figure 1 and online supplemental eTable 2). The cumulative incidence of neurodevelopmental disorders by age across different gestational age categories is shown as online supplemental eFigure 2. Children were followed to a mean age of 14.6 years (range: 10.0–19.8).

Maternal and child descriptive characteristics are presented by gestational age (table 1). As expected, compared with children born full-term, those born between 34^+0^ and 38^+6^ weeks were more likely to be born to older mothers, with higher BMI, who continued smoking during pregnancy. These children were also less likely to be born via vaginal delivery, more likely to be twins and had mothers who were nulliparous and experienced pregnancy-related complications such as diabetes or PCOS, as well as a history of mental health issues. Moreover, these children were more likely to be male, were more likely to be born small or large for gestational age and had lower 5-min APGAR scores.

**Table 1:** Descriptive characteristics of study population (N=1 036 664)

Variables	Weeks of gestation				
	34^+0^ to 40^+6^	34^+0^ to 35^+6^	36^+0^ to 37^+6^	38^+0^ to 38^+6^	39^+0^ to 40^+6^
N	1 036 664	21 087	79 435	143 096	793 046
Maternal factors					
Maternal age (years)					
<20	25 954 (2.50)	583 (2.76)	2040 (2.57)	3268 (2.28)	20 063 (2.53)
20–24	164 819 (15.90)	3079 (14.60)	11 913 (15.00)	20 818 (14.55)	129 009 (16.27)
25–29	329 008 (31.74)	6250 (29.64)	24 037 (30.26)	43 603 (30.47)	255 118 (32.17)
30–34	324 420 (31.29)	6524 (30.94)	24 658 (31.04)	45 202 (31.59)	248 036 (31.28)
≥35	192 463 (18.57)	4651 (22.06)	16 787 (21.13)	30 205 (21.11)	140 820 (17.76)
Maternal body mass index (kg/m^2^)					
<18.5	21 947 (2.12)	542 (2.57)	1858 (2.34)	3348 (2.34)	16 199 (2.04)
18.5–24.9	362 200 (34.94)	7041 (33.39)	26 339 (33.16)	48 043 (33.57)	280 777 (35.4)
25.0–29.9	125 435 (12.10)	2550 (12.09)	9151 (11.52)	16 797 (11.74)	96 937 (12.22)
30–34	46 390 (4.47)	1041 (4.94)	3850 (4.85)	6877 (4.81)	34 622 (4.37)
≥35	22 007 (2.12)	523 (2.48)	2021 (2.54)	3690 (2.58)	15 773 (1.99)
Missing	458 685 (44.25)	9390 (44.53)	36 216 (45.59)	64 341 (44.96)	348 738 (43.97)
Smoking during pregnancy					
Never	858 137 (82.78)	16 971 (80.48)	64 545 (81.26)	117 889 (82.38)	658 732 (83.06)
Stopped during first trimester	38 411 (3.71)	718 (3.40)	2795 (3.52)	4742 (3.31)	30 156 (3.8)
Continued	115 409 (11.13)	2751 (13.05)	9923 (12.49)	16 796 (11.74)	85 939 (10.84)
Missing	24 707 (2.38)	647 (3.07)	2172 (2.73)	3669 (2.56)	18 219 (2.3)
Mode of delivery					
Vaginal	795 403 (76.73)	11 823 (56.07)	53 876 (67.82)	102 424 (71.58)	627 280 (79.1)
Instrumental	74 360 (7.17)	1020 (4.84)	4663 (5.87)	8019 (5.60)	60 658 (7.65)
Planned C-section	77 261 (7.45)	2392 (11.34)	8423 (10.60)	19 823 (13.85)	46 623 (5.88)
Other C-section	89 258 (8.61)	5846 (27.72)	12 443 (15.66)	12 786 (8.94)	58 183 (7.34)
Missing data	382 (0.04)	6 (0.03)	30 (0.04)	44 (0.03)	302 (0.04)
Multiple births					
Singletons	1 008 794 (97.31)	15 663 (74.28)	67 359 (84.80)	135 626 (94.78)	790 146 (99.63)
Twins	27 870 (2.69)	5231 (24.81)	12 049 (15.17)	7470 (5.22)	2900 (0.37)
Triplets	(0.00)	193 (0.92)	27 (0.03)	(0.00)	0 (0)
Marital status					
Married, cohabiting	929 109 (89.62)	18 527 (87.86)	70 404 (88.63)	128 454 (89.77)	711 724 (89.75)
Not married, not cohabiting	104 940 (10.12)	2479 (11.76)	8803 (11.08)	14 285 (9.98)	79 373 (10.01)
Missing data	2615 (0.25)	81 (0.38)	228 (0.29)	357 (0.25)	1949 (0.25)
Parity					
0	420 069 (40.52)	10 982 (52.08)	34 835 (43.85)	53 697 (37.53)	320 555 (40.42)
≥1	616 160 (59.44)	10 088 (47.84)	44 568 (56.11)	89 333 (62.43)	472 171 (59.54)
Missing data	435 (0.04)	17 (0.08)	32 (0.04)	66 (0.05)	320 (0.04)
Diabetes status					
None	880 329 (84.92)	17 508 (83.03)	64 281 (80.92)	117 208 (81.91)	681 332 (85.91)
Type 1 DM	5473 (0.53)	798 (3.78)	2471 (3.11)	1397 (0.98)	807 (0.1)
Type 2 DM	8268 (0.80)	281 (1.33)	1201 (1.51)	1779 (1.24)	5007 (0.63)
Gestational DM	142 594 (13.76)	2500 (11.86)	11 482 (14.45)	22 712 (15.87)	105 900 (13.35)
PCOS					
No	1 028 254 (99.19)	20 799 (98.63)	78 558 (98.9)	141 710 (99.03)	787 187 (99.26)
Yes	8410 (0.81)	288 (1.37)	877 (1.10)	1386 (0.97)	5859 (0.74)
Psychiatric diagnosis in inpatient care					
No	1 026 753 (99.04)	20 772 (98.51)	78 457 (98.77)	141 607 (98.96)	785 917 (99.1)
Yes	9911 (0.96)	315 (1.49)	978 (1.23)	1489 (1.04)	7129 (0.9)
Psychotropic medication (N05 or 06)					
No	1 006 133 (97.05)	20 322 (96.37)	76 647 (96.49)	138 361 (96.69)	770 803 (97.2)
Yes	30 531 (2.95)	765 (3.63)	2788 (3.51)	4735 (3.31)	22 243 (2.8)
Hypertensive disorders					
Chronic hypertension	10 686 (1.03)	527 (2.50)	1461 (1.84)	1924 (1.34)	6774 (0.85)
Gestational hypertension	14 757 (1.42)	501 (2.38)	1806 (2.27)	2655 (1.86)	9795 (1.24)
Pre-eclampsia	20 894 (2.02)	2300 (10.91)	4996 (6.29)	3992 (2.79)	9606 (1.21)
Socioeconomic status					
Upper white-collar	172 951 (16.68)	3526 (16.72)	12 827 (16.15)	23 308 (16.29)	133 290 (16.81)
Lower white-collar	372 030 (35.89)	7571 (35.90)	29 158 (36.71)	52 417 (36.63)	282 884 (35.67)
Blue collar	150 550 (14.52)	3100 (14.7)	11 968 (15.07)	21 128 (14.76)	114 354 (14.42)
Other	181 468 (17.50)	3612 (17.13)	13 720 (17.27)	25 027 (17.49)	139 109 (17.54)
Missing data	159 665 (15.40)	3278 (15.55)	11 762 (14.81)	21 216 (14.83)	123 409 (15.56)
Country of birth					
Finland	974 194 (93.97)	19 822 (94.00)	74 614 (93.93)	134 730 (94.15)	745 028 (93.95)
Other countries	62 470 (6.03)	1265 (6.00)	4821 (6.07)	8366 (5.85)	48 018 (6.05)
Offspring factors					
Offspring year of birth					
1996–2000	271 725 (26.21)	5519 (26.17)	21 716 (27.34)	38 210 (26.7)	206 280 (26.01)
2001–2005	264 403 (25.51)	5285 (25.06)	20 311 (25.57)	37 276 (26.05)	201 531 (25.41)
2006–2010	278 394 (26.85)	5767 (27.35)	20 850 (26.25)	37 236 (26.02)	214 541 (27.05)
2011–2014	222 142 (21.43)	4516 (21.42)	16 558 (20.84)	30 374 (21.23)	170 694 (21.52)
Sex					
M	529 261 (51.05)	11 587 (54.95)	42 869 (53.97)	75 558 (52.8)	399 247 (50.34)
F	507 403 (48.95)	9500 (45.05)	36 566 (46.03)	67 538 (47.2)	393 799 (49.66)
Gestational age at delivery, mean (SD)	39.3 (1.60)	35.0 (0.60)	37.2 (0.60)	38.5 (0.30)	40.3 (0.8)
Birth weight, mean (range 1%–99%)	3464 (2140–4650)	2477 (660–5300)	3012 (1080–6040)	3342 (1420–6675)	3636 (1300–6315)
Head circumference (range 1%–99%)	34.8 (31.0–38.0)	32.4 (23–47.5)	33.8 (23.5–56)	34.6 (22.150.2)	35.2 (23–50.2)
5-min Apgar score					
0–6	9335 (0.9)	884 (4.19)	1241 (1.56)	1172 (0.82)	6038 (0.76)
7–10	509 419 (49.14)	9448 (44.80)	37 940 (47.76)	70 496 (49.26)	391 535 (49.37)
Missing data	517 910 (49.96)	10 755 (51.00)	40 254 (50.68)	71 428 (49.92)	395 473 (49.87)
Birth size for gestational age*					
Small	32 203 (3.11)	2020 (9.58)	5159 (6.49)	5898 (4.12)	19 126 (2.41)
Appropriate	973 217 (93.88)	17 490 (82.94)	69 838 (87.92)	131 069 (91.60)	754 820 (95.18)
Large	30 732 (2.96)	1370 (6.50)	4387 (5.52)	6083 (4.25)	18 892 (2.38)
Missing data	512 (0.05)	207 (0.98)	51 (0.06)	46 (0.03)	208 (0.03)

Data are presented as n (%) unless otherwise indicated.

T1DM was defined using the ICD-10 code E10, and T2DM was identified using the ICD-10 E11, E14 and O24.1, or the use of a glucose-lowering medication other than insulin (ATC code A10B). Gestational DM was defined using the ICD-10 O24.4. PCOS was identified using ICD-10 E28.2 and N97.0 and ICD-9 256.4 and 628.0. Psychiatric diagnoses were defined using ICD-10 F00–F99, ICD-9 290–319 and ICD-8 290–317. Psychotropic medication use was identified based on dispensation of medications within ATC code groups N05 (antipsychotics, anxiolytics, hypnotics and sedatives) or N06 (antidepressants and stimulants). Chronic hypertension was identified using ICD-10 I10–I13 and O10 and ICD-9 401.0, 401.1, 401.9, 402.0, 402.1, 402.9, 403.0, 403.1, 403.9, 404.0, 404.1 and 404.9. Gestational hypertension was defined using ICD-10 O13 and ICD-9 642.4 and 642.9. Pre-eclampsia was identified using ICD-10 O11 and O14 and ICD-9 642.4, 642.5 and 642.7.

* Small and large are defined as birth weight and/or birth length, above or below the two SDs from the mean for gestation and sex-specific in the Finnish population,^1^ based on the International Societies of Pediatric Endocrinology and the Growth Hormone Research Society.^2^

DM, diabetes mellitus; PCOS, polycystic ovary syndrome.

### Risk of neurodevelopmental disorders in children between 34^+0^ and 35^+6^ weeks

**Figure 2:**
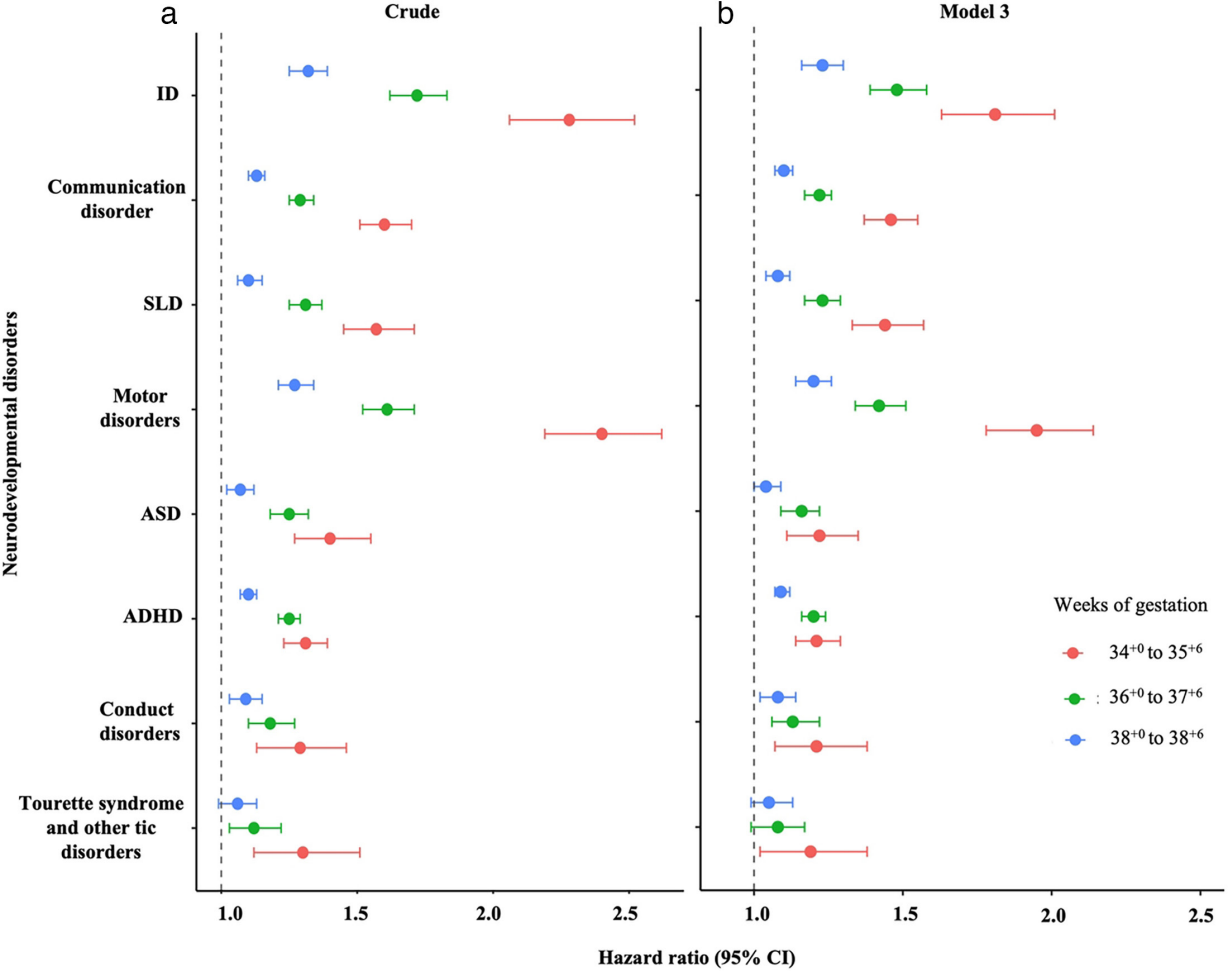
Association between gestational age at birth and risk of neurodevelopmental disorders during childhood and adolescence from a Finnish population cohort of children born between 1994 and 2014. HRs along with 95% CI are presented for (**a**) unadjusted estimates and (**b**) estimates from model 3, which adjusts for the child’s year of birth, parity, maternal age at the child’s birth, socioeconomic status, mode of delivery, maternal smoking during pregnancy, maternal hypertensive disorders, maternal diabetes, maternal BMI, PCOS, maternal history of psychiatric disorders and birth size. Children born at full term (39^+0^ to 40^+6^) were considered as the reference group (HR=1). F70–F79: intellectual disabilities (ID); F80: communication disorders; F81: specific learning disorders (SLD); F82: motor disorders; F84: autism spectrum disorder (ASD); F90: attention-deficit/ hyperactivity disorder (ADHD); F91: conduct disorders and F95: Tourette syndrome and other tic disorders.

Children born between 34^+0^ and 35^+6^ weeks had a higher risk of all neurodevelopmental disorders studied compared with full-term children (figure 2 and online supplemental eTable 3). The highest risk was observed for motor disorders, with a crude HR of 2.40 (95% CI 2.19 to 2.62), followed by a higher risk for ID (HR=2.28 (95% CI 2.06 to 2.52)).

These children also had a higher risk of communication disorders (HR=1.60 (95% CI 1.51 to 1.70)) and SLD (HR=1.57 (95% CI 1.45 to 1.71)), along with ASD (HR=1.40 (95% CI 1.27 to 1.54)), ADHD (HR=1.31 (95% CI 1.23 to 1.39)), and conduct disorders (HR=1.29 (95% CI 1.13 to 1.46)). Moreover, the risk for Tourette syndrome and other tic disorders was also higher (HR=1.30 (95% CI 1.12 to 1.51)) for those born between 34^+0^ and 35^+6^ weeks. After adjusting for potential confounders, these associations were slightly attenuated, yet the risk remained statistically significant. For example, after full adjustment (ie, model 3), the HR for ID decreased to 1.81 (95% CI 1.63 to 2.01) and for motor disorders to 1.95 (95% CI 1.78 to 2.14) (figure 2 and online supplemental eTable 4). However, when the Apgar score was included in model 4, the effect estimates remained largely unchanged relative to model 3, suggesting a limited or negligible role in these associations (online supplemental eTable 5).

### Risk of neurodevelopmental disorders in children born between 36^+0^ and 37^+6^ weeks

Similar to children born between 34^+0^ and 35^+6^ weeks, children born between 36^+0^ and 37^+6^ weeks also showed a higher risk for all neurodevelopmental disorders examined compared with full-term children (figure 2 and online supplemental eTable 3). The greatest risk was found for ID, with a crude HR of 1.72 (95% CI 1.62 to 1.83), followed closely by a higher risk for motor disorders (HR=1.61 (95% CI 1.52 to 1.71)).

These children were also at a higher risk for SLD (HR 1.31 (95% CI 1.25 to 1.37)), communication disorders (HR 1.29 (95% CI 1.25 to 1.34)), ASD (HR 1.25 (95% CI 1.18 to 1.32)), ADHD (HR 1.25 (95% CI 1.22 to 1.29)), conduct disorders (HR 1.18 (95% CI 1.10 to 1.27)), and Tourette syndrome and other tic disorders (HR 1.12 (95% CI 1.03 to 1.22)) compared with full-term children. After adjusting for potential confounders, all observed associations remained statistically significant, though with some attenuation in effect sizes. For example, after full adjustment (ie, model 3), the HR for ID decreased to 1.60 (95% CI 1.50 to 1.71) and for motor disorders to 1.48 (95% CI 1.40 to 1.57) (figure 2 and online supplemental eTable 4). Again, Apgar score adjustment did not influence these associations (online supplemental eTable 5).

### Risk of neurodevelopmental disorders in children born at 38^+0^ to 38^+6^ weeks

Children born at 38^+0^ to 38^+6^ weeks had a slightly higher risk for several neurodevelopmental disorders compared with those born full-term (figure 2 and online supplemental eTable 3). The highest risks were observed for ID, with a crude HR of 1.32 (95% CI 1.24 to 1.39), followed by motor disorders (HR=1.27 (95% CI 1.21 to 1.34)).

These children also had a higher risk of communication disorders (HR 1.13 (95% CI 1.10 to 1.16)), SLD (HR 1.10 (95% CI 1.06 to 1.15)), ADHD (HR 1.10 (95% CI 1.07 to 1.02)), and conduct disorders (HR 1.09 (95% CI 1.03 to 1.15)), with ASD showing a borderline significant crude association (HR 1.07 (95% CI 1.02 to 1.12)). The risk for Tourette syndrome and other tic disorders was not statistically significant (HR 1.06 (95% CI 0.99 to 1.13)). After adjustment for potential confounders, most associations remained statistically significant, although the effect sizes were slightly reduced. The association with ASD, however, did not remain significant in the fully adjusted model (model 3). For example, the adjusted HR for ASD was reduced to 1.04 (95% CI 0.99 to 1.09) (figure 2 and online supplemental eTable 4). Similar to the results observed in the other gestational age groups, adding the Apgar score in model 4 did not alter the associations (online supplemental eTable 5).

### Sensitivity analysis

The sensitivity analysis incorporating 99% CIs (online supplemental eTable 6) produced results consistent with the above analyses (figure 2 and online supplemental eTable 3).

### Sex-specific differences

**Figure 3:**
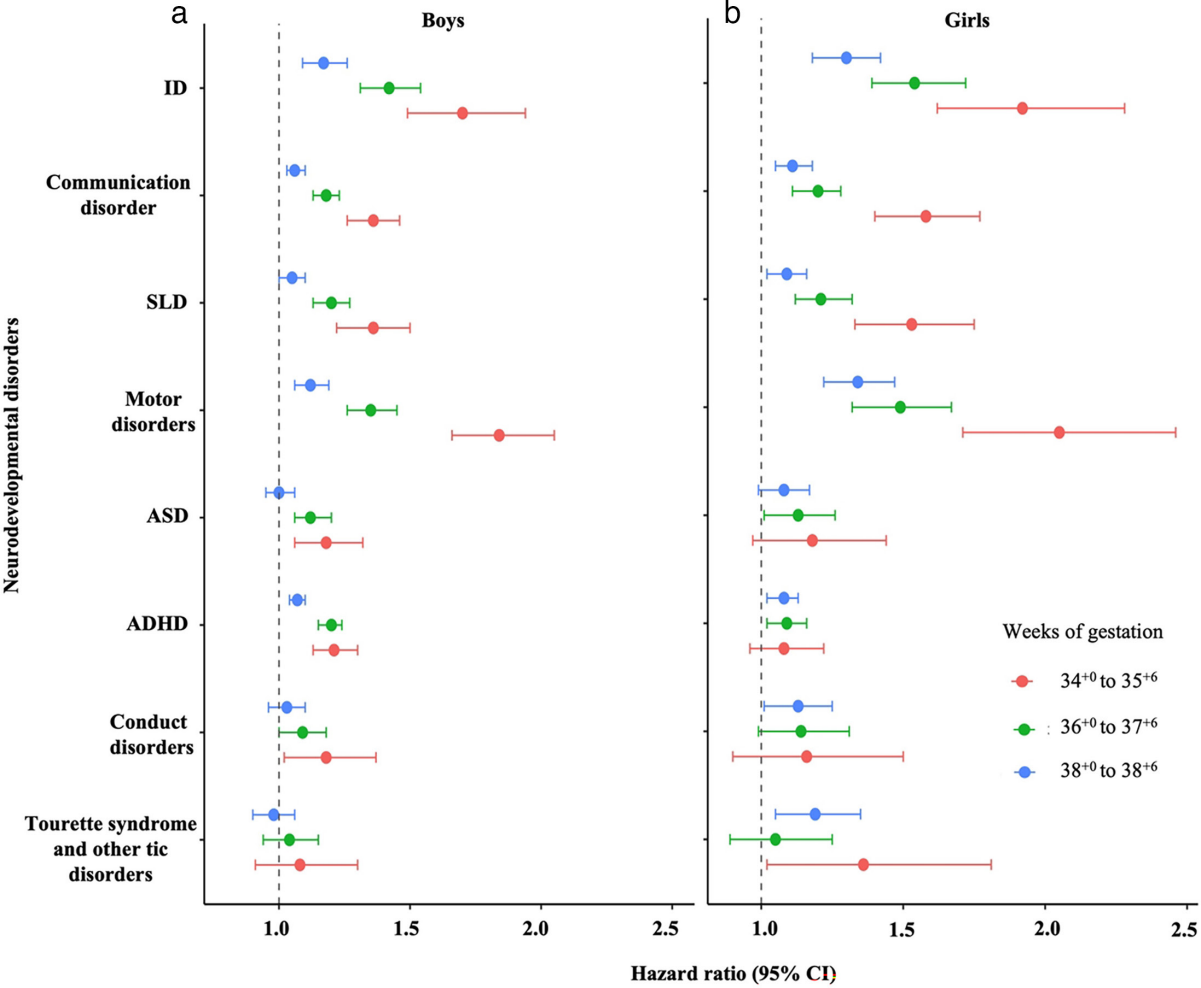
Association between gestational age at birth and risk of neurodevelopmental disorders during childhood and adolescence in (**a**) boys and (**b**) girls from a Finnish population cohort of children born between 1994 and 2014. HRs are adjusted using model 3, which accounts for potential confounders, including the child’s year of birth, parity, maternal age at the child’s birth, socioeconomic status, mode of delivery, maternal smoking during pregnancy, maternal hypertensive disorders, maternal diabetes, maternal BMI, PCOS, maternal history of psychiatric disorders and birth size. Children born at full term (39^+0^ to 40^+6^) were considered as the reference group. F70–F79: intellectual disabilities (ID); F80: communication disorders; F81: specific learning disorders (SLD); F82: motor disorders; F84: autism spectrum disorder (ASD); F90: attention-deficit/ hyperactivity disorder (ADHD); F91: conduct disorders and F95: Tourette syndrome and other tic disorders.

Multiplicative interactions between gestational age groups and sex were statistically significant for all neurodevelopmental disorders, suggesting potential effect modification (online supplemental eTable 7). Despite this, the patterns of observed associations were broadly similar in boys and girls, with overlapping CIs across all outcomes (figure 3 and online supplemental eTables 8 and 9).

### Sibling-pair analysis

We used a sibling-pair comparison design to investigate whether associations between the exposure (ie, born between 34^+0^ and 35^+6^ weeks or between 36^+0^ and 37^+6^ weeks) and neurodevelopmental disorders could be explained by unmeasured familial confounding, such as shared genetic and environmental factors.

### Risk of neurodevelopmental disorders in sibling-pair comparisons for children born between 34^+0^ and 35^+6^ weeks

**Figure 4:**
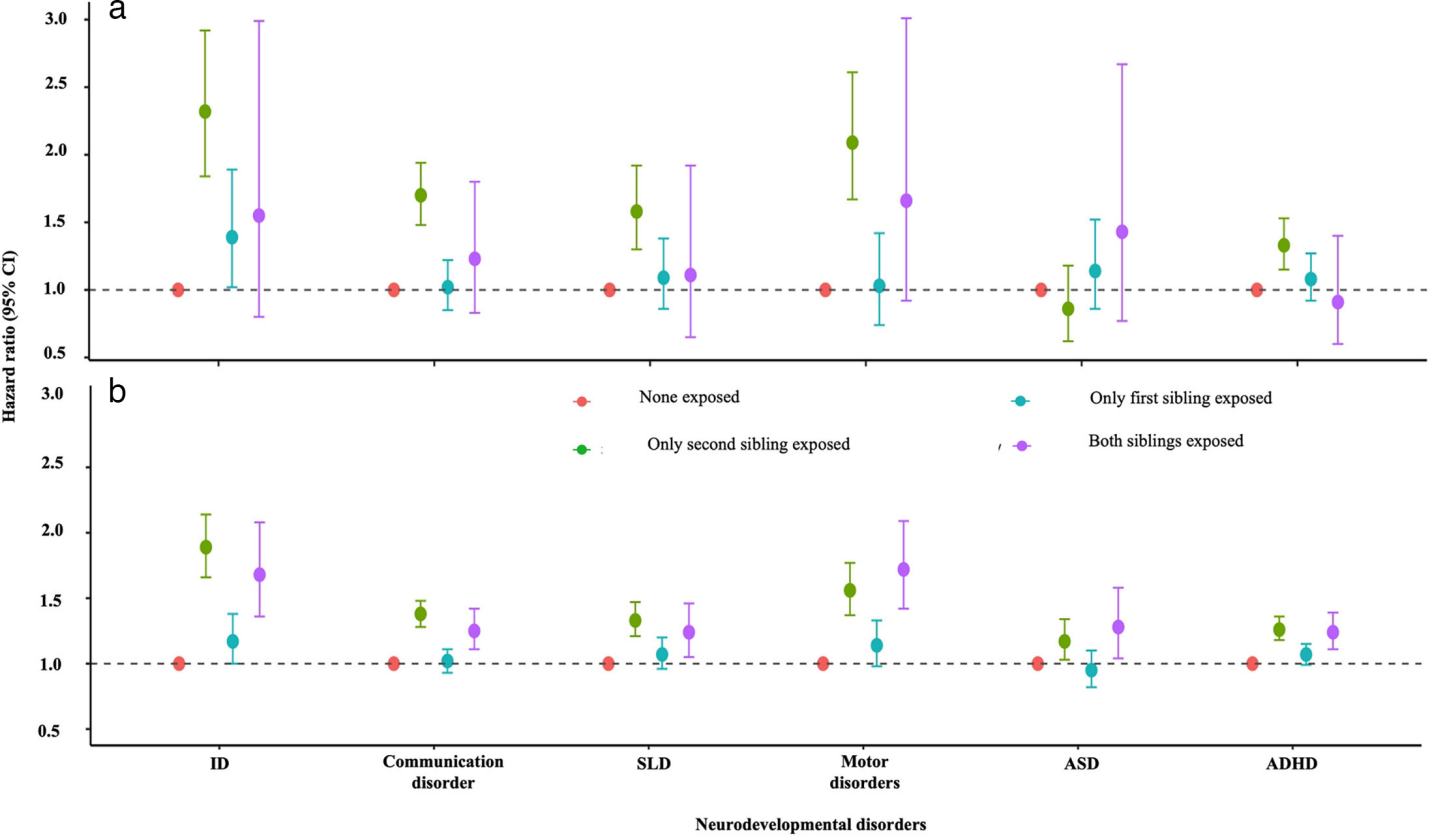
Association between gestational age at birth and risk of neurodevelopmental disorders in the second sibling of the pair (model 5) within a Finnish population cohort of children born between 1994 and 2014, followed until 2021: (a) children born at 34^+0^ to 35^+6^ weeks and (b) children born at 36^+0^ to 37^+6^ weeks. Sibling pairs with no sibling exposed were used as a reference. A difference between green and blue HRs indicates an association not completely explained by within-pair shared familial factors. The wider purple error bars indicate a smaller number of pairs with both siblings being exposed. Models are adjusted for the child’s year of birth, parity, maternal age at the child’s birth, socioeconomic status, mode of delivery, maternal smoking during pregnancy, maternal hypertensive disorders, maternal diabetes, maternal BMI, PCOS, maternal history of psychiatric disorders, birth size, corresponding ICD F-diagnosis in the first sibling and interpregnancy interval length (model 5). F70–F79: intellectual disabilities (ID); F80: communication disorders; F81: specific learning disorders (SLD); F82: motor disorders; F84: autism spectrum disorder (ASD); F90: attention-deficit/ hyperactivity disorder (ADHD); F91: conduct disorders and F95: Tourette syndrome and other tic disorders.

The second (younger) sibling had higher risks for certain neurodevelopmental disorders in those pairs in which only the second sibling was exposed, that is, born at 34^+0^ to 35^+6^ weeks, compared with those pairs where only the first (older) sibling was exposed. This indicates that the detected associations between being born in weeks 34^+0^ to 35^+6^ and offspring disorders were not completely because of shared familial factors (model 5; figure 4 and online supplemental eTable 10). Specifically, for communication disorders, the second siblings had an HR of 1.70 (95% CI 1.48 to 1.94) in pairs where only the second sibling was exposed, compared with 1.02 (95% CI 0.85 to 1.22) in pairs where only the first sibling was exposed. Similarly, for motor disorders, the HR of the second siblings was 2.09 (95% CI 1.67 to 2.61) when only second siblings were exposed versus 1.03 (95% CI 0.74 to 1.42) when only first siblings were exposed, and for SLD, the corresponding HR was 1.58 (95% CI 1.30 to 1.92) compared with 1.09 (95% CI 0.86 to 1.38).

### Risk of neurodevelopmental disorders in sibling-pair comparisons for children born between 36^+0^ and 37^+6^ weeks

Similarly, as for births at 34^+0^ to 35^+6^ weeks, the second siblings born had a higher risk for certain neurodevelopmental disorders if born at 36^+0^ to 37^+6^ weeks (exposed) than if only their younger sibling was exposed (model 5; figure 4 and online supplemental eTable 11). In particular, for ID, the HR for the second sibling was 1.89 (95% CI 1.66 to 2.14) in pairs where only the second sibling was exposed, compared with 1.17 (95% CI 1.00 to 1.38) in pairs where only the first sibling was exposed. For motor disorders, the corresponding HRs were 1.56 (95% CI 1.37 to 1.77) versus 1.14 (95% CI 0.98 to 1.33); for ADHD, 1.26 (95% CI 1.18 to 1.36) versus 1.07 (95% CI 0.99 to 1.15) and for SLD, 1.33 (95% CI 1.21 to 1.47) compared with 1.07 (95% CI 0.96 to 1.20), indicating that the associations were not fully explained by shared familial factors.

## Discussion

Using a large nation-wide population-based study of more than 1 million children born between 34**^+^**^0^ and 40**^+^**^6^ weeks’ gestation, we found that those born late-preterm as well as those born at 38^+0^ to 38^+6^ weeks had higher risks of developing neurodevelopmental disorders compared to full-term children (39^+0^ to 40^+6^ weeks’ gestation). The elevated risks were mainly seen for ID, motor disorders, communication disorders, and SLD, with HRs ranging from 1.57 to 2.40 among children born between 34^+0^ and 38^+6^ weeks. We also observed higher risks for ASD, ADHD, conduct disorders, and Tourette syndrome, although the effect estimates for these conditions were smaller (HRs 1.29–1.40). Importantly, these associations were largely independent of socioeconomic, maternal and birth factors, except for ASD and Tourette syndrome, and did not differ significantly between boys and girls. Further, by using sibling-pair comparisons, we found that the association between gestational age at birth and certain neurodevelopmental disorders, such as communication disorders, motor disorders, and SLD, is likely not completely explained by shared familial genetic or environmental factors, proposing a potential causal relationship.

We found that children born late-preterm and early-term had higher risks of communication disorders and SLD (eg, reading and arithmetic) and motor disorders compared with those born full-term, with the risk decreasing linearly as gestational age increased. For example, those born between 34**^+^**^0^ and 35**^+^**^6^ weeks had a 2.4-fold risk for motor disorders, whereas those born between 36**^+^**^0^ and 37**^+^**^6^ and at 38 weeks had 1.6-fold and 1.3-fold risks, respectively. Importantly, these observations were only mildly attenuated and remained statistically significant across different models accounting for potential confounders. Our findings are consistent with prior prospective studies based on smaller cohorts that have reported higher odds of motor and language delays, as well as reading and mathematical difficulties, in early and middle childhood among children born late-preterm or early-term.^19 27 28^ Moreover, our findings of higher risks for motor, SLD, and communication disorders are in line with a large nation-wide Swedish study, which reported similar elevated effect estimates among late-preterm or early-term children.^14^ However, the Swedish study classified motor impairments by combining cerebral palsy with developmental coordination disorder and grouped cognitive impairments such as ID, SLD, and communication disorders into a single composite category. In contrast, our study examined these diagnostic categories separately, allowing for a more nuanced interpretation. What is novel in this study, beyond examining separate neurodevelopmental diagnoses in a large nation-wide cohort, is the longer follow-up period and the persistence of the observed risks across both the 34**^+^**^0^ and 35**^+^**^6^ weeks and 36**^+^**^0^ and 37**^+^**^6^ gestational age groups, even in sibling-pair comparisons.

Consistent with previous studies,^1 4 12 17^ we observed that children born between 34**^+^**^0^ and 37^+6^ weeks were at higher risk for ASD and ADHD. However, we did not observe an elevated risk of ASD among those born at 38 weeks. This finding contrasts with earlier studies and may be explained by the fact that previous studies often pooled children born at 37 and 38 weeks into a single category. From a developmental perspective, it is well established that each additional week of gestation contributes approximately 4.5 cm^3^ of brain volume, even within the term window.^29^ Our findings suggest that combining 37 and 38 weeks into a single early-term category may obscure meaningful differences in neurodevelopmental risk and highlight the importance of examining these gestational ages separately.

In this study, we also observed that those born between 34**^+^**^0^ and 38^+6^ weeks were at higher risk for ID, consistent with previous population-based studies,^13 14^ and importantly, this association was independent of shared familial and genetic factors. Although our findings align with prior large-scale registry-based research, they contradict results from a US-based prospective population cross-sectional study of 5946 births,^5^ which reported cognitive impairment only among those born before 34 weeks, as assessed via the National Institutes of Health Toolbox, and found these effects to be independent of genetic influences. There are several possible reasons for these differences in findings. Registry-based studies typically include much larger samples, offering greater statistical power to detect smaller effect sizes. They capture a broader population, whereas prospective cohorts may under-represent higher-risk groups due to selection bias. Differences in follow-up duration also matter; registry data may reveal later-emerging difficulties not yet evident during the cohort’s assessment period.

The exact mechanism underlying the elevated risk of neurodevelopmental disorders among late-preterm and early-term children remains largely unknown. Most neurodevelopmental disorders, such as ASD and ADHD, have a strong genetic component. However, not all risk can be attributed to hereditary, leaving room for environmental influences, including biological factors. In our study, children born before 38 weeks showed higher risks for all neurodevelopmental disorders examined. Importantly, the risk for ID, communication disorders, motor disorders, and SLD persisted even after accounting for shared familial factors through sibling-pair analysis, suggesting that biological vulnerability related to earlier gestational age may play a greater role than genetic predisposition alone. One plausible explanation lies in the critical brain maturation that occurs during the third trimester of pregnancy. During this period, the fetal brain undergoes rapid and complex development, including neurogenesis, neuronal migration, synaptogenesis and onset of myelination.^30^ These processes are critical for establishing large-scale brain networks, such as ‘rich-club hubs’, which play a major role in higher-order cognitive and behavioural functioning. Between 30 and 40 weeks of gestation, there is a substantial increase in the number of connections between rich-club regions and the rest of the cortex.^31^ Earlier birth may interrupt the development and integration of these networks, thereby increasing the risk of neurodevelopmental disturbances. In addition, late-preterm children may be more vulnerable to acute perinatal insults, such as hypoxia or metabolic disturbances, including neonatal hypoglycaemia, which can compromise oxygen and nutrient delivery to the developing brain and further disrupt typical brain development.

The present study has some important clinical implications. Children born at weeks 34–36 make up most of the preterm population, and they are often considered ‘low-risk’ for neurodevelopmental concerns. Our population-based findings indicate clinically meaningful increases in neurodevelopmental risk even within this gestational window, raising questions about the conventional use of 37 weeks as the threshold for full maturity. For example, children born at 34^+0^ to 35^+6^ weeks had a prevalence of any neurodevelopmental disorder of 21.6%, compared with 15.0% among those born at 39^+0^ to 40^+6^ weeks, corresponding to an absolute increase of 6.6%. At the population level, this translates to ~1370 additional affected children in the Finnish birth cohort. Similarly, children born at 36^+0^ to 37^+6^ weeks had a 19.0% prevalence (absolute increase 4%), corresponding to ~3230 additional cases, whereas those born at 38 weeks had a 16.0% prevalence (absolute increase 1%), or ~1350 additional cases. These data highlight that even modest absolute differences can result in a substantial number of additional affected children when considered across the population. Importantly, these risks span across multiple developmental domains, such as motor, communication and SLD, which are often under-recognised in late-preterm and early-term children. Although most children born in the late-preterm and early-term period do well, our findings support enhanced developmental monitoring and earlier intervention in these gestational groups, even when neonatal outcomes appear reassuring. From a public health perspective, the gradient in risk between 34^+0^ and 38^+6^ weeks suggests that extending gestation by even 1 or 2 weeks may meaningfully reduce the population burden of neurodevelopmental disorders.

### Strengths and limitations

To our knowledge, this is one of the largest studies to focus specifically on the associations between late-preterm and early-term births and a broader range of neurodevelopmental disorders, particularly those that have received less research attention. The main strengths of the study are its large, population-based design with long-term follow-up into late adolescence. Neurodevelopmental diagnoses were obtained from high-quality validated national health registries.^32^ Importantly, the cohort includes children born after 1995, reflecting more contemporary obstetric and neonatal care practices. Gestational age was categorised in a fine-grained manner to capture subtle differences across the late-preterm and early-term spectrum. Neurodevelopmental disorders that are often overlooked in this population, such as motor and communication disorders, were included among those assessed. The associations between gestational age at birth and risk for neurodevelopmental disorders were adjusted for a range of detailed familial and perinatal factors drawn from multiple registries. Finally, by incorporating sibling-pair comparisons, the study was able to address potential unmeasured shared familial confounding, strengthening the case for an independent effect of gestational age.

The study has some limitations that should be considered when interpreting the results. We had no information on neonatal complications such as neonatal hypoglycaemia, which may contribute to later neurodevelopmental disorders. The large population-based design improves generalisability, but maternal behaviours like smoking during pregnancy may be under-reported, introducing attribution bias. Although we accounted for several shared maternal risk factors for earlier birth, time-varying confounders between pregnancies such as maternal stress or infection could not be addressed. The sibling-pair design adjusts for shared familial and environmental influences but does not eliminate the effect of sibling-specific genetic variation. Limited statistical power in the exposure-discordant sibling analysis made it difficult to examine some outcomes, for example, conduct disorders. Reliance on medical records and healthcare service use may have resulted in underascertainment of children with milder (-2 SD to -1 SD) cognitive or motor impairments. Residual confounding from unmeasured factors, including paternal genetic liability, may still be present. As we assessed several neurodevelopmental outcomes, we attempted to correct for potential type I error. Therefore, we conducted a sensitivity analysis using 99% CI, which yielded results consistent with the original analysis. Finally, the findings should also be interpreted in the context of the Finnish healthcare system and population, which may limit their applicability to more diverse or other than Nordic populations.

In summary, we found a higher risk of childhood neurodevelopmental disorders among those born between 34**^+^**^0^ and 38^+6^ weeks compared with full-term births. Interestingly, elevated risks were observed for several understudied disorders, including the communication disorders, motor disorders, and SLD, with associations largely independent of unmeasured sibling-shared familial factors. These gestational age groups have traditionally received less clinical attention, often being considered low risk. Our findings underscore the importance of recognising that births within the late-preterm and early-term range also carry a measurable risk for neurodevelopmental disorders. Our results also highlight the value of promoting longer gestation, reinforcing the notion that every additional week in utero may support more optimal neurodevelopmental outcomes.

## Supplementary material

10.1136/bmjph-2025-003708 online supplemental file 1
