# Supplementary material for: Neurodevelopmental risks of late-preterm and early-term births: a population-based study from Finland
Source: BMJ Public Health. 2026 Feb 27;4(1):e003708. doi: 10.1136/bmjph-2025-003708 (PMC12958876; doi:10.1136/bmjph-2025-003708)
Supplement: online supplemental file 1 [file bmjph-4-1-s001.docx]

**Supplementary Materials**

**Neurodevelopmental Risks of Late-Preterm and Early-Term Births: A Population-Based Study from Finland**

Samson Nivins, PhD^a^, Mika Gissler PhD^b,c,d,e,f^, Catharina Lavebratt MSc, PhD^b,c,†^

^a^ Department of Women’s and Children’s Health, Karolinska Institutet, Stockholm, Sweden

^b^ Center for Molecular Medicine, Karolinska University Hospital, Stockholm, Sweden

^c^ Department of Molecular Medicine and Surgery, Karolinska Institutet, Stockholm, Sweden

^d^ Department of Data and Analytics, Finnish Institute for Health and Welfare, Helsinki, Finland.

^e^ Research Centre for Child Psychiatry, University of Turku, Turku, Finland

^f^Academic Primary Health Care Centre, Region Stockholm, Stockholm, Sweden

Corresponding author: Dr Samson Nivins**,** Woman and Children’s Health, Karolinska Institute, Stockholm, Sweden **[**[samson.nivins@ki.se](mailto:samson.nivins@ki.se)].

**eResults**

The week-by-week gestation analysis showed that risk for most neurodevelopmental disorders was highest at 34 and 35 weeks, decreased at 36 and 37 weeks, and were lowest at 38 weeks. For example, the adjusted HRs for communication disorders, SLD, and motor disorders decreased from 1.47 to 1.10, 1.52 to 1.08, and 2.17 to 1.20, respectively **(eTable 1).** These patterns supported the use of grouped gestational categories in subsequent analyses.

**eTable 1** Association analysis between gestational age at birth, stratified by each gestational week, and neurodevelopmental disorders in the overall cohort between 34^+0^ to 38^+6^

| **Neurodevelopmental disorders** | **Weeks of gestation** | | | | |
| --- | --- | --- | --- | --- | --- |
|  | **34** | **35** | **36** | **37** | **38** |
| Intellectual disabilities | 1.72 (1.45-2.04) | 1.86 (1.64-2.12) | 1.43 (1.28-1.59) | 1.51 (1.40-1.63) | 1.23 (1.16-1.30) |
| Communication disorders | 1.47 (1.34-1.63) | 1.45 (1.34-1.57) | 1.27 (1.20-1.36) | 1.19 (1.14-1.25) | 1.10 (1.07-1.13) |
| Specific learning disorder | 1.52 (1.34-1.73) | 1.39 (1.26-1.55) | 1.26 (1.16-1.37) | 1.21 (1.15-1.28) | 1.08 (1.04-1.12) |
| Motor disorders | 2.17 (1.90-2.49) | 1.82 (1.61-2.04) | 1.42 (1.28-1.57) | 1.43 (1.33-1.53) | 1.20 (1.14-1.26) |
| ASD | 1.19 (1.02-1.39) | 1.24 (1.09-1.40) | 1.10 (1.00-1.21) | 1.18 (1.11-1.26) | 1.04 (1.00-1.09) |
| ADHD | 1.18 (1.07-1.30) | 1.23 (1.14-1.32) | 1.24 (1.18-1.31) | 1.19 (1.14-1.23) | 1.09 (1.07-1.12) |
| Conduct disorders | 1.12 (0.91-1.39) | 1.27 (1.08-1.49) | 1.12 (0.99-1.26) | 1.14 (1.05-1.24) | 1.08 (1.02-1.14) |
| Tourette Syndrome and other tic disorders | 1.01 (0.78-1.32) | 1.30 (1.08-1.56) | 1.02 (0.88-1.19) | 1.10 (1.00-1.22) | 1.05 (0.99-1.13) |

Data are presented as adjusted hazard ratios (HRs) and (95% CI). Abbreviations: ASD, autism spectrum disorder; and ADHD, attention-deficit/hyperactivity disorder.

The model was adjusted for the child’s year of birth, parity, maternal age at child’s birth, socioeconomic status, mode of delivery, maternal smoking during pregnancy, maternal hypertensive disorders, maternal diabetes, maternal BMI, PCOS, maternal history of psychiatric disorders, and birth size. Children born 39^+0^ to 40^+6^ weeks were considered as reference group.

**eTable 2** Number of participants with neurodevelopmental disorders among children born between 34^+0^ to 40^+6^

| **Neurodevelopmental disorders (ICD codes)** | **Weeks of gestation** | | | | |
| --- | --- | --- | --- | --- | --- |
|  | **34^+0^ to 40^+6^** | **34^+0^ to 35^+6^** | **36^+0^ to 37^+6^** | **38** | **39^+0^ to 40^+6^** |
| N | 1036664 | 21087 | 79435 | 143096 | 793046 |
| Intellectual disabilities (F70-79) | 9735 (0.94) | 390 (1.85) | 1129 (1.42) | 1569 (1.10) | 6647 (0.84) |
| Communication disorders (F80) | 35586 (3.43) | 1066 (5.06) | 3300 (4.15) | 5251 (3.67) | 25969 (3.27) |
| Specific learning disorder (F81) | 20945 (2.02) | 609 (2.89) | 1963 (2.47) | 3030 (2.12) | 15343 (2.64) |
| Motor disorders (F82) | 12019 (1.16) | 609 (2.89) | 1963 (2.47) | 3030 (2.12) | 15343 (1.93) |
| ASD (F84) | 16103 (1.55) | 425 (2.02) | 1464 (1.84) | 2288 (1.60) | 11926 (1.50) |
| ADHD (F90) | 46639 (4.50) | 1137 (5.39) | 4233 (5.33) | 6798 (4.75) | 34471 (4.35) |
| Conduct disorders (F91) | 10100 (0.97) | 244 (1.16) | 869 (1.09) | 1461 (1.02) | 7526 (0.95) |
| Tourette Syndrome and other Tic Disorders (F95) | 7190 (0.69) | 177 (0.84) | 591 (0.74) | 1021 (0.71) | 5401 (0.68) |

Data are presented as n (%) unless otherwise indicated. Abbreviations: ASD, autism spectrum disorders; ADHD, attention-deficit hyperactivity disorders.

**eTable 3** Association between gestational age at birth and neurodevelopmental disorders among children between 34^+0^ to 40^+6^ (Crude model with Risk difference)

| **Neurodevelopmental disorders** | **Weeks of gestation** | | | | | |
| --- | --- | --- | --- | --- | --- | --- |
|  | **34^+0^ to 35^+6^** | | **36^+0^ to 37^+6^** | | **38** | |
|  | **RD (%)** | **HR (95%CI)** | **RD (%)** | **HR (95%CI)** | **RD (%)** | **HR (95%CI)** |
| Intellectual disabilities | 1.01 | 2.28 (2.06-2.52) | 0.58 | 1.72 (1.62-1.83) | 0.26 | 1.32 (1.25-1.39) |
| Communication disorders | 1.78 | 1.60 (1.51-1.70) | 0.88 | 1.29 (1.25-1.34) | 0.39 | 1.13 (1.10-1.16) |
| Specific learning disorder | 1.38 | 1.57 (1.45-1.71) | 0.61 | 1.31 (1.25-1.37) | 0.27 | 1.10 (1.06-1.15) |
| Motor disorders | 0.51 | 2.40 (2.19-2.62) | 0.34 | 1.61 (1.52-1.71) | 0.10 | 1.27 (1.21-1.34) |
| ASD | 1.05 | 1.40 (1.27-1.55) | 0.98 | 1.25 (1.18-1.32) | 0.40 | 1.07 (1.02-1.12) |
| ADHD | 0.21 | 1.31 (1.23-1.39) | 0.14 | 1.25 (1.21-1.29) | 0.07 | 1.10 (1.07-1.13) |
| Conduct disorders | 0.16 | 1.29 (1.13-1.46) | 0.06 | 1.18 (1.10-1.27) | 0.03 | 1.09 (1.03-1.15) |
| Tourette Syndrome and other tic disorders | 0.95 | 1.30 (1.12-1.51) | 0.54 | 1.12 (1.03-1.22) | 0.18 | 1.06 (0.99-1.13) |

Data are presented as RD, risk difference; and HR, hazard ratios and (95% CI). Abbreviations: ASD, autism spectrum disorders; ADHD, attention-deficit hyperactivity disorders. Children born 39^+0^ to 40^+6^ weeks were considered as reference group.

**eTable 4** Association between gestational age at birth and neurodevelopmental disorders among children between 34^+0^ to 40^+6^ (adjusted models)

| **Neurodevelopmental disorders** | **Weeks of gestation** | | | | | | | | |
| --- | --- | --- | --- | --- | --- | --- | --- | --- | --- |
|  | **Model 1** | | | **Model 2** | | | **Model 3** | | |
|  | **34^+0^ to 35^+6^** | **36^+0^ to 37^+6^** | **38** | **34^+0^ to 35^+6^** | **36^+0^ to 37^+6^** | **38** | **34^+0^ to 35^+6^** | **36^+0^ to 37^+6^** | **38** |
| Intellectual disabilities | 2.07 (1.86-2.29) | 1.64 (1.54-1.75) | 1.28 (1.21-1.35) | 2.03 (1.83-2.25) | 1.60 (1.50-1.71) | 1.26 (1.19-1.33) | 1.81 (1.63-2.01) | 1.48 (1.39-1.58) | 1.23 (1.16-1.30) |
| Communication disorders | 1.54 (1.45-1.64) | 1.28 (1.23-1.32) | 1.13 (1.09-1.16) | 1.51 (1.42-1.61) | 1.25 (1.20-1.29) | 1.11 (1.07-1.14) | 1.46 (1.37-1.55) | 1.22 (1.17-1.26) | 1.10 (1.07-1.13) |
| Specific learning disorder | 1.54 (1.42-1.68) | 1.30 (1.24-1.36) | 1.11 (1.06-1.15) | 1.50 (1.38-1.63) | 1.26 (1.20-1.32) | 1.08 (1.04-1.13) | 1.44 (1.33-1.57) | 1.23 (1.17-1.29) | 1.08 (1.04-1.12) |
| Motor disorders | 2.17 (1.99-2.38) | 1.54 (1.45-1.63) | 1.24 (1.18-1.31) | 2.07 (1.89-2.27) | 1.48 (1.40-1.57) | 1.21 (1.16-1.28) | 1.95 (1.78-2.14) | 1.42 (1.34-1.51) | 1.20 (1.14-1.26) |
| ASD | 1.30 (1.18-1.43) | 1.22 (1.16-1.29) | 1.07 (1.02-1.12) | 1.25 (1.13-1.38) | 1.17 (1.11-1.24) | 1.05 (1.00-1.10) | 1.22 (1.11-1.35) | 1.16 (1.09-1.22) | 1.04 (1.00-1.09) |
| ADHD | 1.28 (1.21-1.36) | 1.26 (1.22-1.30) | 1.12 (1.09-1.15) | 1.24 (1.17-1.31) | 1.22 (1.18-1.26) | 1.10 (1.07-1.13) | 1.21 (1.14-1.29) | 1.20 (1.16-1.24) | 1.09 (1.07-1.12) |
| Conduct disorders | 1.27 (1.11-1.44) | 1.18 (1.10-1.27) | 1.11 (1.05-1.17) | 1.22 (1.08-1.39) | 1.14 (1.06-1.22) | 1.08 (1.02-1.14) | 1.21 (1.07-1.38) | 1.13 (1.06-1.22) | 1.08 (1.02-1.14) |
| Tourette Syndrome and other tic disorders | 1.22 (1.05-1.42) | 1.11 (1.02-1.21) | 1.07 (1.00-1.15) | 1.19 (1.02-1.38) | 1.08 (0.99-1.17) | 1.05 (0.98-1.13) | 1.19 (1.02-1.38) | 1.08 (0.99-1.17) | 1.05 (0.99-1.13) |

Data are presented as adjusted hazard ratios (HRs) and (95% CI). Abbreviations: ASD, autism spectrum disorder; and ADHD, attention-deficit/hyperactivity disorder.

**Model 1:** Adjusted for the child’s year of birth, parity, maternal age at child’s birth, socioeconomic status, and mode of delivery.

**Model 2**: Adjusted for the variables in Model 1, with additional adjustment for maternal smoking during pregnancy, maternal hypertensive disorders, maternal diabetes, maternal BMI, PCOS, and maternal history of psychiatric disorders.

**Model 3:** Adjusted for the variables in Model 2 with additional adjustment for birth size

Children born 39^+0^ to 40^+6^ weeks were considered as reference group for all the models.

**eTable 5** Adjustment for Apgar 5 minutes score: the association between gestational age at birth and neurodevelopmental disorders among children between 34^+0^ to 40^+6^

| **Neurodevelopmental disorders** | **Weeks of gestation** | | |
| --- | --- | --- | --- |
|  | **34^+0^ to 35^+6^** | **36^+0^ to 37^+6^** | **38** |
| Intellectual disabilities | 1.81 (1.63-2.01) | 1.48 (1.39-1.58) | 1.23 (1.16-1.30) |
| Communication disorders | 1.46 (1.37-1.55) | 1.22 (1.17-1.26) | 1.10 (1.07-1.13) |
| Specific learning disorder | 1.44 (1.33-1.57) | 1.23 (1.17-1.29) | 1.08 (1.04-1.12) |
| Motor disorders | 1.96 (1.78-2.14) | 1.42 (1.34-1.51) | 1.20 (1.14-1.26) |
| ASD | 1.22 (1.11-1.35) | 1.16 (1.09-1.22) | 1.04 (1.00-1.09) |
| ADHD | 1.21 (1.14-1.29) | 1.20 (1.16-1.24) | 1.09 (1.07-1.12) |
| Conduct disorders | 1.21 (1.06-1.38) | 1.13 (1.06-1.22) | 1.08 (1.02-1.14) |
| Tourette Syndrome and other tic disorders | 1.19 (1.02-1.38) | 1.08 (0.99-1.17) | 1.05 (0.99-1.13) |

Data are presented as adjusted hazard ratios (HRs) and (95% CI). Abbreviations: ASD, autism spectrum disorder; and ADHD, attention-deficit/hyperactivity disorder. The model was adjusted for the child’s year of birth, parity, maternal age at child’s birth, socioeconomic status, mode of delivery, maternal smoking during pregnancy, maternal hypertensive disorders, maternal diabetes, maternal BMI, PCOS, maternal history of psychiatric disorders, birth size, along with Apgar scores at five minutes (Model 4). Children born 39^+0^ to 40^+6^ weeks were considered as reference group.

**eTable 6** Sensitivity analysis of results in eTable 3 Model 3: Association analysis at α=1% (99% Confidence interval) between gestational age at birth and neurodevelopmental disorders among children between 34^+0^ to 40^+6^ (Model 3)

| **Neurodevelopmental disorders** | **Weeks of gestation** | | |
| --- | --- | --- | --- |
|  | **34^+0^ to 35^+6^** | **36^+0^ to 37^+6^** | **38** |
| Intellectual disabilities | 1.81 (1.58-2.07) | 1.48 (1.36-1.62) | 1.23 (1.14-1.32) |
| Communication disorders | 1.46 (1.35-1.58) | 1.22 (1.16-1.28) | 1.10 (1.06-1.14) |
| Specific learning disorder | 1.44 (1.29-1.61) | 1.23 (1.15-1.31) | 1.08 (1.02-1.13) |
| Motor disorders | 1.95 (1.73-2.20) | 1.42 (1.32-1.54) | 1.20 (1.12-1.28) |
| ASD | 1.22 (1.07-1.39) | 1.16 (1.07-1.24) | 1.04 (0.98-1.11) |
| ADHD | 1.21 (1.12-1.31) | 1.2 (1.15-1.26) | 1.09 (1.06-1.13) |
| Conduct disorders | 1.21 (1.02-1.44) | 1.13 (1.03-1.25) | 1.08 (1.00-1.16) |
| Tourette Syndrome and other tic disorders | 1.19 (0.97-1.45) | 1.08 (0.96-1.21) | 1.05 (0.97-1.15) |

Data are presented as adjusted hazard ratios (HRs) and (99% CI). Abbreviations: ASD, autism spectrum disorder; and ADHD, attention-deficit/hyperactivity disorder. The model was adjusted for the child’s year of birth, parity, maternal age at child’s birth, socioeconomic status, mode of delivery, maternal smoking during pregnancy, maternal hypertensive disorders, maternal diabetes, maternal BMI, PCOS, maternal history of psychiatric disorders, and birth size. Children born 39^+0^ to 40^+6^ weeks were considered as reference group.

**eTable 7** Analysis of interaction between gestational age groups and sex on the risk of neurodevelopmental disorders

| **Neurodevelopmental disorders** | **34^+0^ to 35^+6^ weeks** | | **36^+0^ to 37^+6^ weeks** | | **38 weeks** | |
| --- | --- | --- | --- | --- | --- | --- |
|  | **Chi-square value (*X^2^)*** | **P value** | **Chi-square value (*X^2^)*** | **P value** | **Chi-square value (*X^2^)*** | **P value** |
| Intellectual disabilities | 8.90 | 0.003 | 40.10 | <.0001 | 61.90 | <.0001 |
| Communication disorders | 111.47 | <.0001 | 490.24 | <.0001 | 776.18 | <.0001 |
| Specific learning disorder | 29.72 | <.0001 | 148.18 | <.0001 | 226.79 | <.0001 |
| Motor disorders | 73.68 | <.0001 | 210.33 | <.0001 | 281.20 | <.0001 |
| ASD | 72.70 | <.0001 | 251.53 | <.0001 | 366.03 | <.0001 |
| ADHD | 209.71 | <.0001 | 791.84 | <.0001 | 1116.59 | <.0001 |
| Conduct disorders | 41.70 | <.0001 | 144.20 | <.0001 | 230.72 | <.0001 |
| Tourette Syndrome and other tic disorders | 8.67 | 0.003 | 31.81 | <.0001 | 63.04 | <.0001 |

The *X^2^* represents the multiplicative interaction term between gestational age group and sex. The Model was unadjusted for any confounders.

Abbreviations: ASD, autism spectrum disorder; and ADHD, attention-deficit/hyperactivity disorder.

**eTable 8** Number of boys and girls with neurodevelopmental disorders among children born between 34^+0^ to 40^+6^

| **Neurodevelopmental disorders** | **Weeks of gestation** | | | | |
| --- | --- | --- | --- | --- | --- |
|  | **34^+0^ to 40^+6^** | **34^+0^ to 35^+6^** | **36^+0^ to 37^+6^** | **38** | **39^+0^ to 40^+6^** |
|  | **Boys** | | | | |
| N | 529261 | 11587 | 42869 | 75558 | 399247 |
| Intellectual disabilities | 6108 (1.15) | 241 (2.08) | 709 (1.65) | 977 (1.29) | 4181 (1.05) |
| Communication disorders | 25386 (4.8) | 751 (6.48) | 2409 (5.62) | 3777 (5) | 18449 (4.62) |
| Specific learning disorder | 13496 (2.55) | 392 (3.38) | 1302 (3.04) | 1980 (2.62) | 9822 (2.46) |
| Motor disorders | 8859 (1.67) | 376 (3.25) | 972 (2.27) | 1361 (1.8) | 6150 (1.54) |
| ASD | 11680 (2.21) | 319 (2.75) | 1087 (2.54) | 1658 (2.19) | 8616 (2.16) |
| ADHD | 33455 (6.32) | 861 (7.43) | 3176 (7.41) | 4933 (6.53) | 24485 (6.13) |
| Conduct disorders | 7295 (1.38) | 182 (1.57) | 638 (1.49) | 1051 (1.39) | 5424 (1.36) |
| Tourette Syndrome and other tic disorders | 5263 (0.99) | 127 (1.1) | 445 (1.04) | 725 (0.96) | 3966 (0.99) |
|  | **Girls** | | | | |
| N | 507403 | 9500 | 36566 | 67538 | 393799 |
| Intellectual disabilities | 3627 (0.71) | 149 (1.57) | 420 (1.15) | 592 (0.88) | 2466 (0.63) |
| Communication disorders | 10200 (2.01) | 315 (3.32) | 891 (2.44) | 1474 (2.18) | 7520 (1.91) |
| Specific learning disorder | 7449 (1.47) | 217 (2.28) | 661 (1.81) | 1050 (1.55) | 5521 (1.4) |
| Motor disorders | 3160 (0.62) | 135 (1.42) | 344 (0.94) | 528 (0.78) | 2153 (0.55) |
| ASD | 4423 (0.87) | 106 (1.12) | 377 (1.03) | 630 (0.93) | 3310 (0.84) |
| ADHD | 13184 (2.6) | 276 (2.91) | 1057 (2.89) | 1865 (2.76) | 9986 (2.54) |
| Conduct disorders | 2805 (0.55) | 62 (0.65) | 231 (0.63) | 410 (0.61) | 2102 (0.53) |
| Tourette Syndrome and other tic disorders | 1927 (0.38) | 50 (0.53) | 146 (0.4) | 296 (0.44) | 1435 (0.36) |

Data are presented as n (%) unless otherwise indicated. Abbreviations: ASD, autism spectrum disorders; ADHD, attention-deficit hyperactivity disorders.

**eTable 9** Association between gestational age at birth and neurodevelopmental disorders in boys and girls born between 34^+0^ to 40^+6^

| **Neurodevelopmental disorders** | **Weeks of gestation** | | | | | |
| --- | --- | --- | --- | --- | --- | --- |
|  | **Boys** | | | **Girls** | | |
|  | **34^+0^ to 35^+6^** | **36^+0^ to 37^+6^** | **38** | **34^+0^ to 35^+6^** | **36^+0^ to 37^+6^** | **38** |
| Intellectual disabilities | 1.70 (1.49-1.94) | 1.42 (1.31-1.54) | 1.17 (1.09-1.26) | 1.92 (1.62-2.28) | 1.54 (1.39-1.72) | 1.30 (1.18-1.42) |
| Communication disorders | 1.36 (1.26-1.46) | 1.18 (1.13-1.23) | 1.06 (1.03-1.10) | 1.58 (1.40-1.77) | 1.20 (1.11-1.28) | 1.11 (1.05-1.18) |
| Specific learning disorder | 1.36 (1.22-1.50) | 1.20 (1.13-1.27) | 1.05 (1.00-1.10) | 1.53 (1.33-1.75) | 1.21 (1.12-1.32) | 1.09 (1.02-1.16) |
| Motor disorders | 1.84 (1.66-2.05) | 1.35 (1.26-1.45) | 1.12 (1.06-1.19) | 2.05 (1.71-2.46) | 1.49 (1.32-1.67) | 1.34 (1.22-1.47) |
| ASD | 1.18 (1.06-1.32) | 1.12 (1.06-1.20) | 1.00 (0.95-1.06) | 1.18 (0.97-1.44) | 1.13 (1.01-1.26) | 1.08 (0.99-1.17) |
| ADHD | 1.21 (1.13-1.30) | 1.20 (1.15-1.24) | 1.07 (1.04-1.10) | 1.08 (0.96-1.22) | 1.09 (1.02-1.16) | 1.08 (1.02-1.13) |
| Conduct disorders | 1.18 (1.02-1.37) | 1.09 (1.00-1.18) | 1.03 (0.96-1.10) | 1.16 (0.90-1.50) | 1.14 (0.99-1.31) | 1.13 (1.01-1.25) |
| Tourette Syndrome and other tic disorders | 1.08 (0.91-1.30) | 1.04 (0.94-1.15) | 0.98 (0.90-1.06) | 1.36 (1.02-1.81) | 1.05 (0.89-1.25) | 1.19 (1.05-1.35) |

Data are presented as adjusted hazard ratios (HRs) and (95% CI). Abbreviations: ASD, autism spectrum disorder; and ADHD, attention-deficit/hyperactivity disorder. The model was adjusted for the child’s year of birth, parity, maternal age at child’s birth, socioeconomic status, mode of delivery, maternal smoking during pregnancy, maternal hypertensive disorders, maternal diabetes, maternal BMI, PCOS, maternal history of psychiatric disorders, and birth size. Children born 39^+0^ to 40^+6^ weeks were considered as reference group.

**eTable 10** Sibling-pair analysis of gestational age at birth and neurodevelopmental disorders in children born between 34^+0^ to 35^+6^ accounting for unmeasured shared familial confounding

| **Exposure** | **Intellectual disabilities** | **Communication disorders** | **Specific learning disorder** | **Motor disorders** | **ASD** | **ADHD** |
| --- | --- | --- | --- | --- | --- | --- |
| **Crude** |  |  |  |  |  |  |
| Concordant unexposed (neither sibling exposed) | 1 | 1 | 1 | 1 | 1 | 1 |
| Discordant: Exposed second only | 2.37 (1.88-3.00) | 1.76 (1.53-2.02) | 1.61 (1.33-1.95) | 2.11 (1.69-2.64) | 0.88 (0.64-1.22) | 1.39 (1.20-1.60) |
| Discordant: Exposed first only | 1.53 (1.13-2.07) | 1.08 (0.90-1.29) | 1.12 (0.89-1.42) | 1.11 (0.80-1.54) | 1.17 (0.88-1.55) | 1.13 (0.96-1.32) |
| Concordant exposed (both siblings exposed) | 1.66 (0.86-3.21) | 1.31 (0.89-1.93) | 1.20 (0.70-2.08) | 1.81 (1.00-3.27) | 1.40 (0.75-2.62) | 0.94 (0.61-1.44) |
| **Model 5** |  |  |  |  |  |  |
| Concordant unexposed (neither sibling exposed) | 1 | 1 | 1 | 1 | 1 | 1 |
| Discordant: Exposed second only | 2.32 (1.84-2.92) | 1.70 (1.48-1.94) | 1.58 (1.30-1.92) | 2.09 (1.67-2.61) | 0.86 (0.62-1.18) | 1.33 (1.15-1.53) |
| Discordant: Exposed first only | 1.39 (1.02-1.89) | 1.02 (0.85-1.22) | 1.09 (0.86-1.38) | 1.03 (0.74-1.42) | 1.14 (0.86-1.52) | 1.08 (0.92-1.27) |
| Concordant exposed (both siblings exposed) | 1.55 (0.80-2.99) | 1.23 (0.83-1.80) | 1.11 (0.65-1.92) | 1.66 (0.92-3.01) | 1.43 (0.77-2.67) | 0.91 (0.60-1.40) |

Data are presented as adjusted hazard ratios (HRs) and (95% CI). Abbreviations: ASD, autism spectrum disorder; and ADHD, attention-deficit/hyperactivity disorder.

The Model 5 was adjusted for the child’s year of birth, parity, maternal age at child’s birth, socioeconomic status, mode of delivery, maternal smoking during pregnancy, maternal hypertensive disorders, maternal diabetes, maternal BMI, PCOS, maternal history of psychiatric disorders, birth size, inter-pregnancy interval, and presence of corresponding ICD F-diagnosis in the first sibling.

**eTable 11** Sibling-pair analysis of gestational age at birth and neurodevelopmental disorders in children born between 36^+0^ to 37^+6^ accounting for unmeasured shared familial confounding

| **Exposure** | **Intellectual disabilities** | **Communication disorders** | **Specific learning disorder** | **Motor disorders** | **ASD** | **ADHD** |
| --- | --- | --- | --- | --- | --- | --- |
| **Crude** |  |  |  |  |  |  |
| Concordant unexposed (neither sibling exposed) | 1 | 1 | 1 | 1 | 1 | 1 |
| Discordant: Exposed second only | 1.89 (1.67-2.15) | 1.39 (1.29-1.49) | 1.34 (1.22-1.48) | 1.56 (1.37-1.77) | 1.19 (1.05-1.36) | 1.28 (1.19-1.38) |
| Discordant: Exposed first only | 1.23 (1.05-1.45) | 1.05 (0.96-1.15) | 1.08 (0.96-1.21) | 1.18 (1.02-1.38) | 0.97 (0.84-1.12) | 1.09 (1.01-1.18) |
| Concordant exposed (both siblings exposed) | 1.74 (1.41-2.15) | 1.26 (1.11-1.43) | 1.25 (1.06-1.48) | 1.81 (1.49-2.19) | 1.28 (1.04-1.57) | 1.31 (1.17-1.47) |
| **Model 5** |  |  |  |  |  |  |
| Concordant unexposed (neither sibling exposed) | 1 | 1 | 1 | 1 | 1 | 1 |
| Discordant: Exposed second only | 1.89 (1.66-2.14) | 1.38 (1.28-1.48) | 1.33 (1.21-1.47) | 1.56 (1.37-1.77) | 1.17 (1.03-1.34) | 1.26 (1.18-1.36) |
| Discordant: Exposed first only | 1.17 (1.00-1.38) | 1.02 (0.93-1.11) | 1.07 (0.96-1.20) | 1.14 (0.98-1.33) | 0.95 (0.82-1.1) | 1.07 (0.99-1.15) |
| Concordant exposed (both siblings exposed) | 1.68 (1.36-2.08) | 1.25 (1.11-1.42) | 1.24 (1.05-1.46) | 1.72 (1.42-2.09) | 1.28 (1.04-1.58) | 1.24 (1.11-1.39) |

Data are presented as adjusted hazard ratios (HRs) and (95% CI). Abbreviations: ASD, autism spectrum disorder; and ADHD, attention-deficit/hyperactivity disorder.

The Model 5 was adjusted for the child’s year of birth, parity, maternal age at child’s birth, socioeconomic status, mode of delivery, maternal smoking during pregnancy, maternal hypertensive disorders, maternal diabetes, maternal BMI, PCOS, maternal history of psychiatric disorders, birth size, inter-pregnancy interval, and presence of corresponding ICD F-diagnosis in the first sibling.


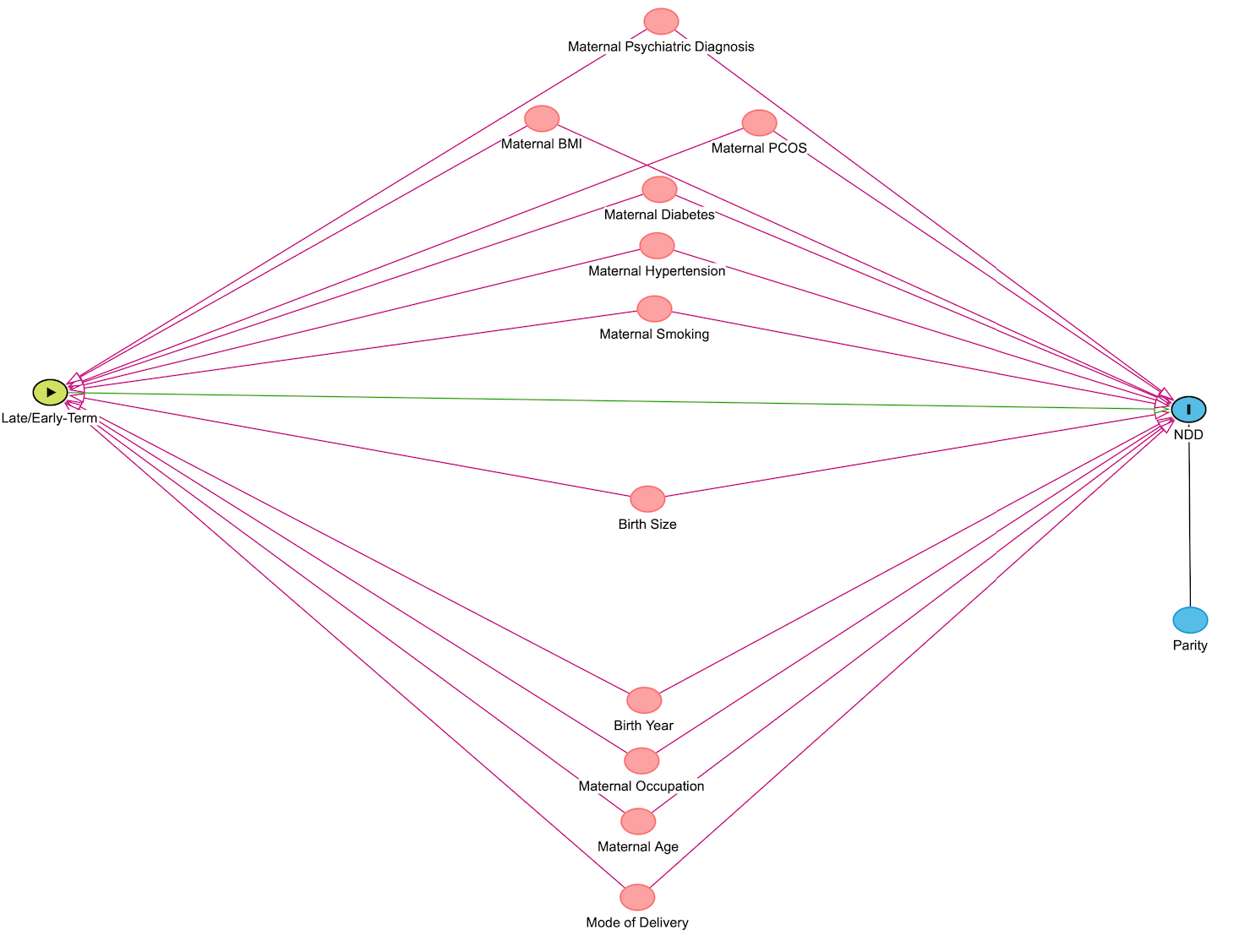


**eFigure 1** Directed acyclic graph (DAG) illustrating the associations between gestational age at birth and the risk of neurodevelopmental disorders (NDDs) during childhood and adolescence. Arrows indicate assumed biasing paths. Dagitty tool (<http://www.dagitty.net>).


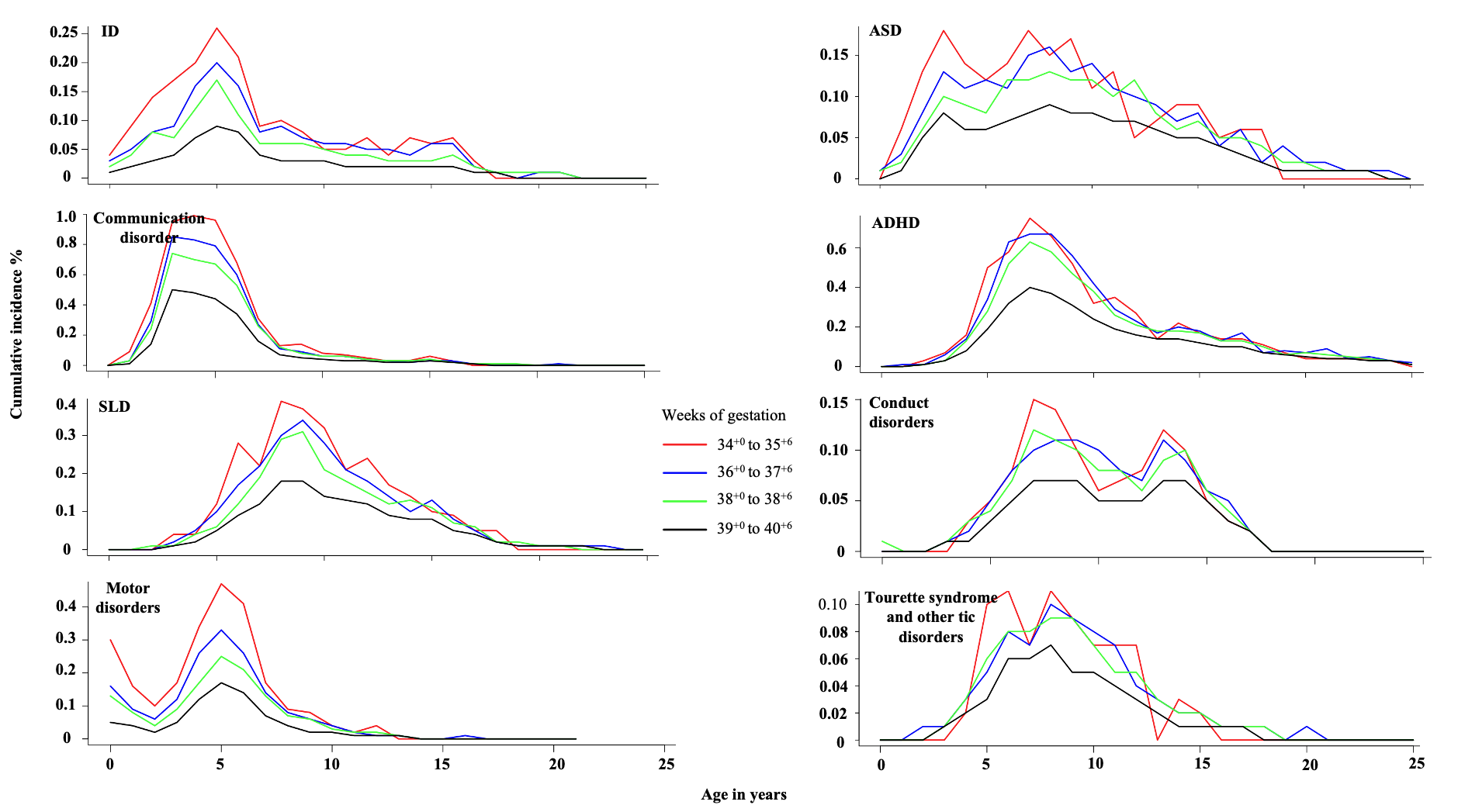


**eFigure 2** The cumulative incidence of neurodevelopmental disorders by age (0 to 25 years) across different gestational age categories.

F70–F79: Intellectual disabilities (ID), F80: communication disorders, F81: specific learning disorders (SLD); F82: motor disorders, F84: autism spectrum disorder (ASD), F90: attention-deficit/ hyperactivity disorder (ADHD), F91: conduct disorders, and F95: Tourette syndrome and other tic disorders.
